# Atrial Fibrillation, Cerebral Amyloid Angiopathy, and Stroke-Related Dysphagia: A Case Report of Gastrointestinal Haemorrhage Following Percutaneous Endoscopic Gastrostomy (PEG) Placement

**DOI:** 10.7759/cureus.88289

**Published:** 2025-07-19

**Authors:** Zain Asif, Hira Zafar, Umar Naveed

**Affiliations:** 1 Acute Stroke Unit, Fairfield General Hospital, Northern Care Alliance, Bury, GBR; 2 Orthopaedics/Internal Medicine, Lak Locum, Manchester, GBR

**Keywords:** atrial fibrillation management, cerebral amyloid angiopathy (caa), general internal medicine, hospice and palliative care, : ischemic stroke, massive gastrointestinal bleeding, percutaneous endoscopic gastrostomy (peg) feeding, post-stroke dysphagia, stroke complications

## Abstract

Atrial fibrillation and cerebral amyloid angiopathy often coexist in older adults and can make post-stroke management particularly challenging, especially when further complicated by comorbidities such as cirrhosis. We present the case of a 78-year-old man admitted with an acute ischaemic stroke and persistent dysphagia. He was initially fed via a nasogastric tube, but ongoing swallowing difficulties led to the insertion of a percutaneous endoscopic gastrostomy (PEG) tube. The patient’s background of atrial fibrillation, cerebral amyloid angiopathy, and cirrhosis created a significant dilemma around the use of anticoagulation and increased his risk of both recurrent stroke and major bleeding. After PEG placement, he developed recurrent gastrointestinal haemorrhage requiring multiple blood transfusions, but no clear source of bleeding was identified on endoscopy or imaging. Despite multidisciplinary management and supportive care, his condition gradually deteriorated, and he died several weeks later, with the precise cause of death remaining uncertain. This case highlights the real-world difficulty of managing anticoagulation in patients with both atrial fibrillation and cerebral amyloid angiopathy, particularly when PEG feeding and other co-morbidities are present. Careful risk assessment and close teamwork are crucial in these complex situations.

## Introduction

Atrial fibrillation (AF) is a common cause of ischaemic stroke in older adults and is typically managed with long-term anticoagulation to reduce the risk of further events [1]. However, in patients with cerebral amyloid angiopathy (CAA), the decision to continue anticoagulation becomes far more complex due to an increased risk of intracerebral and systemic bleeding [2,3]. This overlap between stroke prevention and bleeding risk is becoming increasingly recognised in day-to-day practice.

Dysphagia is another frequent consequence of acute stroke, affecting more than half of patients in the early phase of recovery [4]. While many recover swallowing function over time, some require long-term enteral feeding. Percutaneous endoscopic gastrostomy (PEG) is often used in such cases, but the procedure itself carries risks-particularly in patients with coagulopathies or underlying liver disease. Complications can include infection, aspiration, and gastrointestinal bleeding [5,6].

This case presents the challenge of managing a patient with AF, CAA, and cirrhosis, who required PEG feeding after a severe stroke. The subsequent development of recurrent gastrointestinal bleeding raised difficult questions about how best to balance the competing risks of thrombosis and haemorrhage in a complex, multimorbid patient.

## Case presentation

**Figure 1:**
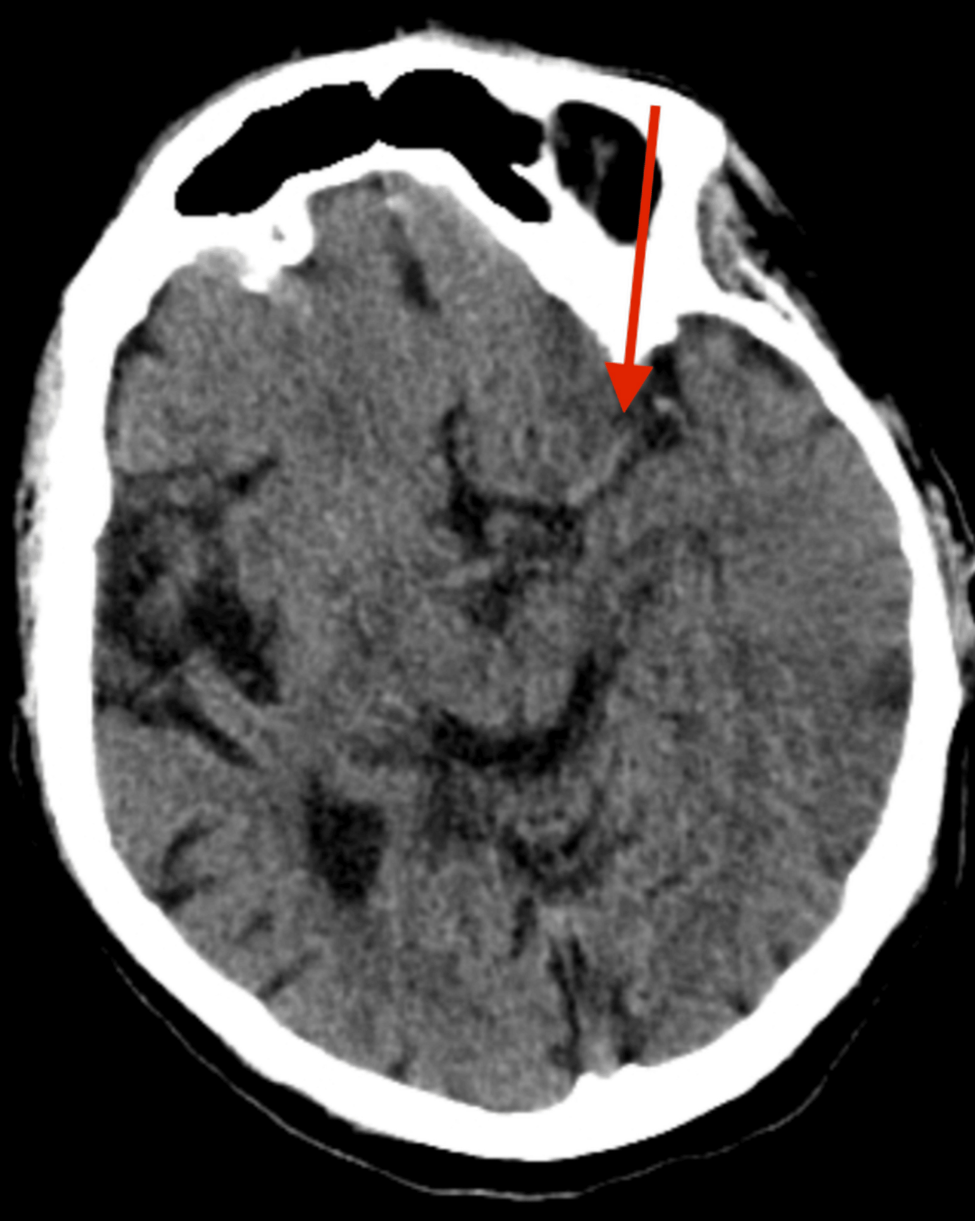
Computed Tomography (CT) Head Showing Left M1 Middle Cerebral Artery (MCA) Hyperdensity Plain CT head showing a hyperdense thrombus (red arrow) within the left M1 segment of the middle cerebral artery. Note the radiological evidence of a prior left frontal infarct.

**Figure 2:**
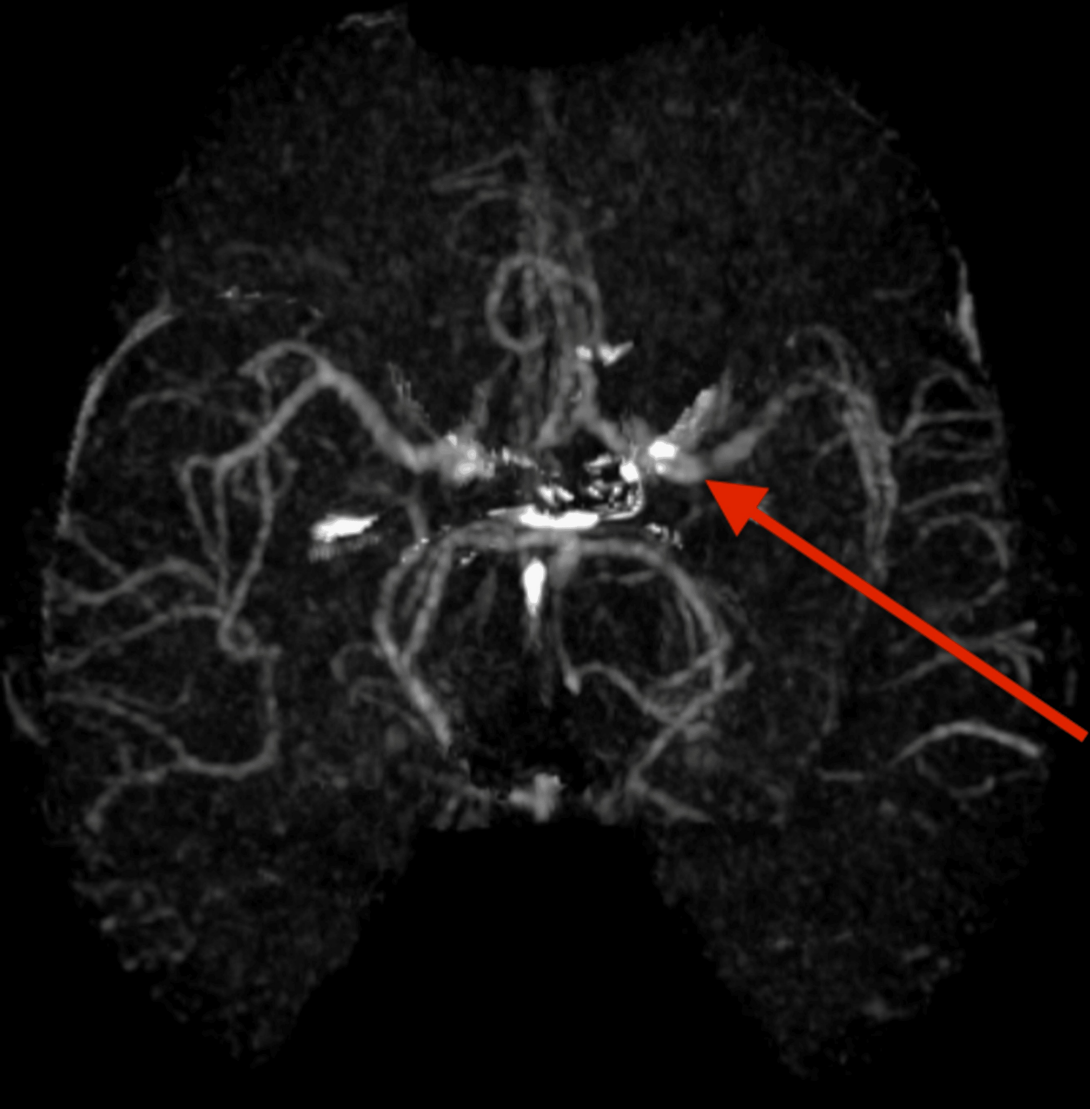
CT Angiography Showing Occlusive Left M1 Middle Cerebral Artery (MCA) Thrombus CT angiogram showing occlusion of the distal left M1 middle cerebral artery (red arrow). Good collateral circulation is seen with retrograde filling of the M2 branches in the left Sylvian fissure.

A 78-year-old man with a history of atrial fibrillation (previously on apixaban but non-adherent for about a year), chronic heart failure, coronary artery calcification, a prior right middle cerebral artery (MCA) territory stroke, chronic kidney disease stage 3, liver cirrhosis, hypertension, rheumatoid arthritis, and other longstanding comorbidities, was admitted in early January with sudden-onset right-sided weakness, right facial droop, drooling, and combined expressive and receptive dysphasia. On initial assessment, he was alert but globally aphasic, with impaired comprehension and speech production. Neurological examination revealed right upper and lower limb weakness of 3/5 power, increased tone, reduced sensation on the right, brisk reflexes in the right upper limb, and a right-sided facial nerve palsy. His vital signs were stable on admission, with no evidence of haemodynamic compromise or hypoxia. Baseline laboratory investigations were broadly consistent with his known chronic kidney disease and mild hepatic dysfunction. His regular medications before admission included apixaban, candesartan, spironolactone, bumetanide, dapagliflozin, dutasteride, tamsulosin, and amlodipine. His pre-morbid Modified Rankin Scale (mRS) score was 1, indicating slight disability before this event, and his National Institutes of Health Stroke Scale (NIHSS) score on admission was 14, reflecting moderate-to-severe neurological impairment. Given his atrial fibrillation with prolonged interruption of anticoagulation, cirrhosis, and concern for cerebral amyloid angiopathy (CAA) on prior imaging, risk assessment was complex: his Congestive Heart Failure, Hypertension, Age ≥75 (2 points), Diabetes Mellitus, Prior Stroke or TIA or Thromboembolism (2 points), Vascular Disease, Age 65-74, Sex Category (Female) (CHA₂DS₂-VASc) score was 7, suggesting a very high annual thromboembolic stroke risk exceeding 9-10% if untreated, while his Hypertension, Abnormal Renal/Liver Function, Stroke, Bleeding History or Predisposition, Labile INR, Elderly (Age >65), Drugs/Alcohol Concomitantly (HAS-BLED) score was 5, indicating a substantial baseline bleeding risk. This highlighted the challenges around anticoagulation in his case. The documented time of symptom onset was around mid-afternoon. An urgent computed tomography (CT) head ruled out intracerebral haemorrhage, and CT angiography showed an occlusive thrombus in the left M1 segment of the middle cerebral artery, likely representing an embolus from atrial fibrillation, with good collateral flow and evidence of an old left frontal infarct, as demonstrated in Figures 1, 2.

**Figure 3:**
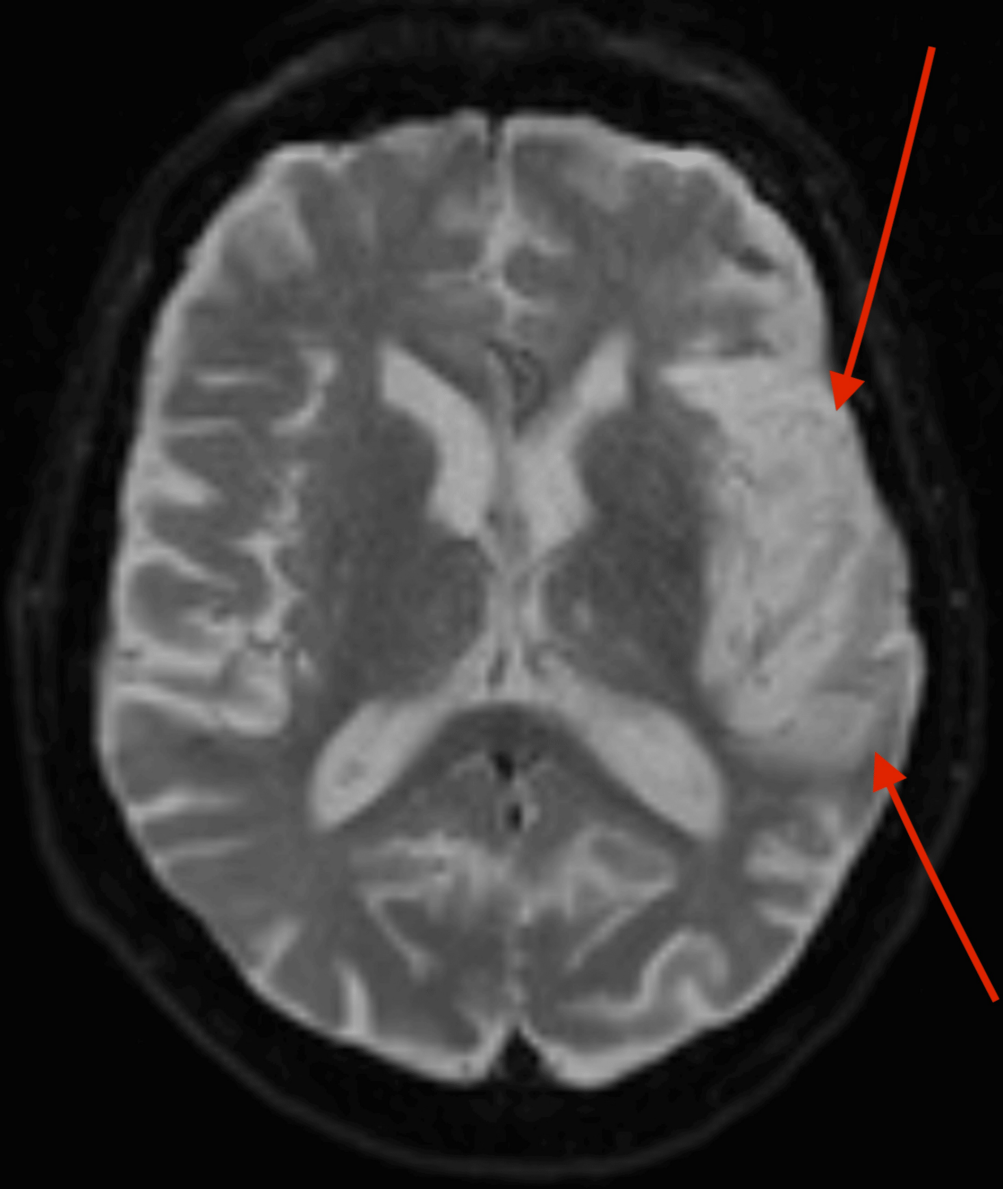
Diffusion-Weighted Magnetic Resonance Imaging (DWI MRI) of the Brain Showing Subacute Infarcts Axial DWI sequence demonstrating large areas of restricted diffusion in the left temporal and frontal lobes (highlighted by arrows), consistent with subacute infarction. These findings, alongside apparent diffusion coefficient (ADC) mapping and other imaging features, supported the diagnosis of cerebral amyloid angiopathy.

He was managed on the acute stroke unit. Due to persistent dysphagia identified by the speech and language therapy team, a nasogastric tube was inserted to provide enteral nutrition. Magnetic resonance imaging (MRI) of the brain was performed to assist with anticoagulation decision-making in the context of his recent stroke, suspected cerebral amyloid angiopathy (CAA), and prior bleeding risk. The scan showed large subacute infarcts in the left temporal and frontal lobes (Figure 3). While this image primarily demonstrated infarction, features supportive of CAA were noted on additional sequences, including multiple cerebral microbleeds and cortical superficial siderosis. These, together with his clinical background and history of previous infarcts, were consistent with probable CAA. He was initially managed conservatively with aspirin 300 mg daily for two weeks, before switching to apixaban 2.5 mg twice daily. His regular antihypertensive and heart failure medications were also continued during admission.

**Figure 4:**
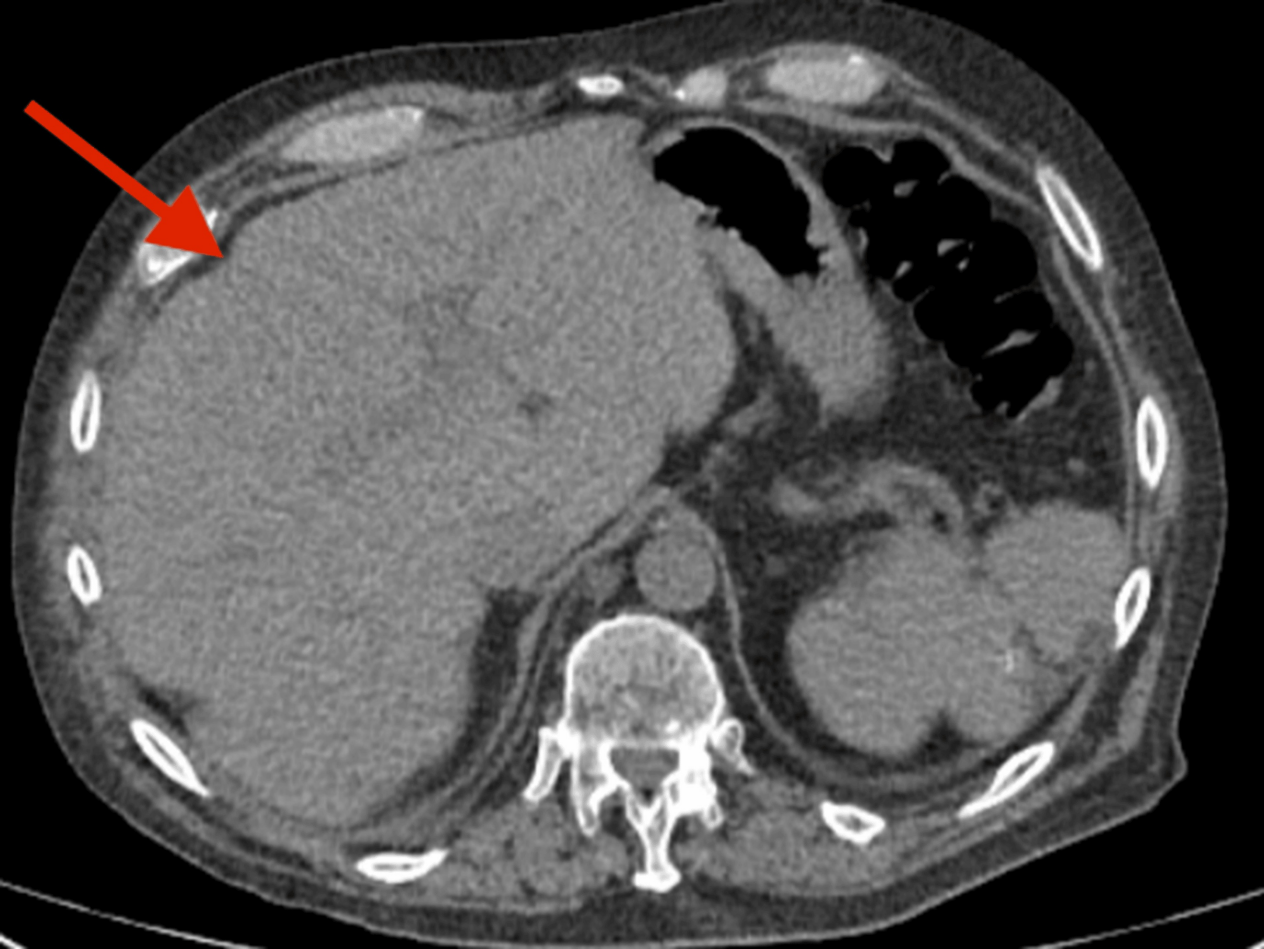
CT Abdomen Showing Cirrhotic Liver and Percutaneous Endoscopic Gastrostomy (PEG) Tube (26/02/2025) CT abdomen with contrast performed on 26 February 2025 showing a cirrhotic liver (red arrow) and an appropriately positioned PEG tube. No signs of active gastrointestinal bleeding were identified.

As swallowing did not improve, a PEG tube was inserted approximately three weeks after admission to provide long-term nutritional support. He was initially managed with aspirin 300 mg daily for two weeks before restarting apixaban at a reduced dose, in line with caution regarding early anticoagulation following a large infarction. Neurologically, he remained alert with a Glasgow Coma Scale (GCS) score of 15, though he continued to have right-sided weakness and global aphasia. The patient remained on apixaban due to his atrial fibrillation and elevated risk of further stroke, but anticoagulation was a concern given his bleeding risk and underlying liver disease. Shortly after PEG placement, he developed recurrent gastrointestinal bleeding, with episodes of melaena and haematemesis. In total, he received nine units of packed red blood cells during admission, with each unit approximately 250-300 mL. The lowest haemoglobin recorded was 76 g/L, which prompted urgent transfusion. Given the absence of any new neurological signs, no repeat head imaging was performed. Haemoglobin levels dropped from 135 g/L on admission to around 100 g/L. These trends, along with changes in renal and liver function, are shown in Table 1. Endoscopic evaluation was performed early, but repeat procedures were at times limited by swallowing difficulties and haemodynamic stability. A CT angiogram of the abdomen was carried out to look for active gastrointestinal bleeding, but it did not demonstrate any contrast extravasation. The underlying cirrhosis was most likely related to chronic metabolic factors, and there was no evidence of portal hypertension on imaging. The PEG tube remained appropriately positioned, and imaging confirmed a cirrhotic liver (Figure 4).

**Table 1:** Serial Blood Results from Admission Onward This table summarises key laboratory results from day 1 to day 69 of admission, including haemoglobin, liver enzymes [alanine aminotransferase (ALT), alkaline phosphatase (ALP)], creatinine, and estimated glomerular filtration rate (eGFR). Reference ranges: haemoglobin 130–170 g/L (male); ALT 10–50 U/L; ALP 30–130 U/L; creatinine 60–110 µmol/L; eGFR >60 mL/min (age-adjusted).

Date	Haemoglobin (g/L)	ALT (U/L)	ALP (U/L)	Creatinine (µmol/L)	eGFR (ml/min)
01/01/25	139	21	179	225	25
03/01/25	135	22	176	217	28
06/01/25	132	26	176	203	35
08/01/25	128	28	179	194	41
10/01/25	129	124	384	166	46
13/01/25	131	109	344	146	50
15/01/25	127	79	301	122	55
20/01/25	127	55	256	112	58
25/01/25	123	36	210	108	60
30/01/25	119	24	227	106	63
05/02/25	109	25	196	102	65
10/02/25	100	21	186	100	66
15/02/25	93	16	178	97	67
20/02/25	76	23	163	95	70
25/02/25	64	21	166	90	71
01/03/25	88	21	153	88	74
05/03/25	96	21	153	87	77
10/03/25	94	18	153	84	73

Blood results showed a gradual improvement in renal function over the admission, while liver enzymes initially rose and later stabilised. Platelets, INR, and electrolytes remained broadly within normal ranges. These ongoing trends are also illustrated in Table 1. Despite ongoing multidisciplinary care, his condition gradually deteriorated, and he died on 30 March 2025. 

The case was referred to the coroner due to concerns over gastrointestinal haemorrhage following gastrostomy placement, and a post-mortem examination was performed. The cause of death was certified as: 1) Upper gastrointestinal bleed; II) left middle cerebral artery infarction, atrial fibrillation, ischaemic heart disease, heart failure, and liver cirrhosis.

## Discussion

This case highlights the complex and often conflicting decisions involved in managing anticoagulation in stroke patients with coexisting atrial fibrillation (AF) and cerebral amyloid angiopathy (CAA). While anticoagulation is a well-established strategy to reduce the risk of cardioembolic stroke in AF [1], the presence of CAA significantly increases the risk of intracerebral haemorrhage [2,3]. The challenge lies in balancing these opposing risks, particularly in patients who are already medically frail or have other bleeding risks.

In this case, cerebral amyloid angiopathy was confirmed radiologically and compounded by a history of cirrhosis, further elevating the risk of bleeding. While guidelines provide general recommendations for stroke prevention in AF, they do not offer detailed direction in the context of confirmed CAA. Some studies have suggested that anticoagulation may be contraindicated in such patients due to high intracranial bleeding risk, but decisions must ultimately be tailored to individual risk profiles [3]. This case underscores the importance of careful, individualised assessment when considering anticoagulation in patients with both cerebral amyloid angiopathy and cirrhosis, recognising that while direct oral anticoagulants (DOACs) may offer advantages over warfarin in many scenarios, their use in this context requires thorough multidisciplinary evaluation given the heightened risks of both thrombosis and major bleeding.

The patient also developed persistent dysphagia following stroke, a recognised complication in more than half of patients in the early phase of recovery [4]. When oral intake cannot be safely maintained, gastrostomy placement is often required. Although percutaneous endoscopic gastrostomy (PEG) is commonly performed, he was ultimately managed with a radiologically inserted gastrostomy (RIG) due to procedural considerations. Gastrostomy placement carries known risks-particularly gastrointestinal bleeding-which are amplified in patients with cirrhosis or those taking anticoagulants [5,6]. In this case, gastrointestinal bleeding followed gastrostomy placement, and no clear source could be identified on imaging, despite multiple investigations. The patient required repeated blood transfusions, and management was complicated by his concurrent need for anticoagulation.

Patients with both atrial fibrillation and CAA are increasingly recognised as particularly high-risk for both thromboembolic and haemorrhagic complications. The Clinical Relevance Of Microbleeds In Stroke (CROMIS-2) study and a recent meta-analysis both highlight how CAA significantly increases the likelihood of intracerebral bleeding recurrence, making anticoagulation decisions even more difficult in this group [7,8]. Although direct oral anticoagulants like apixaban are often preferred over warfarin due to a lower risk of intracranial bleeding, evidence in CAA-specific populations is still limited [8].

For gastrostomy procedures performed while patients remain anticoagulated, bleeding remains a recognised risk, but data from large cohorts suggest it is relatively uncommon-even when anticoagulation is continued [9,10].

This case also underscores the importance of early and sustained multidisciplinary input. Close coordination between stroke physicians, gastroenterologists, and surgical teams was needed to assess feeding tube suitability, manage bleeding episodes, and make complex decisions around anticoagulation in the context of cirrhosis and heightened bleeding risk [11].

Ultimately, despite the best supportive care, the patient’s condition deteriorated, and the cause of death remained unclear. His trajectory reflects a broader reality in stroke medicine, where multiple comorbidities, frailty, and procedural complications converge to create unpredictable outcomes. Case reports such as this may help build an evidence base to support more informed decision-making in similarly complex patients.

## Conclusions

This case highlights the complex and often conflicting considerations involved in managing stroke patients with multiple comorbidities. In individuals with atrial fibrillation and confirmed cerebral amyloid angiopathy, decisions around anticoagulation must be made cautiously, especially when other risk factors such as cirrhosis and the need for enteral feeding are present. This is even more critical given the potential for portal hypertension and varices in cirrhosis, which can further elevate bleeding risk. The development of gastrointestinal bleeding following PEG placement in this context illustrates the real-world challenges faced by clinicians. Multidisciplinary collaboration, individualised risk assessment, and careful monitoring are essential in navigating these high-risk scenarios, where guidelines may offer limited direction. Sharing such cases may help inform clinical decision-making and contribute to a growing body of evidence in a poorly defined area of practice.
